# Analyzing pathways from childhood maltreatment to internalizing symptoms and disorders in children and adolescents (AMIS): a study protocol

**DOI:** 10.1186/s12888-015-0512-z

**Published:** 2015-06-10

**Authors:** Lars O. White, Annette M. Klein, Clemens Kirschbaum, Maria Kurz-Adam, Manfred Uhr, Bertram Müller-Myhsok, Katrin Hoffmann, Susan Sierau, Andrea Michel, Tobias Stalder, Jenny Horlich, Jan Keil, Anna Andreas, Leonhard Resch, Martin J. Binser, Anna Costa, Elena Giourges, Eva Neudecker, Christiane Wolf, Sandra Scheuer, Marcus Ising, Kai von Klitzing

**Affiliations:** 1 Department of Child and Adolescent Psychiatry, Psychotherapy and Psychosomatics, University of Leipzig, Leipzig, Germany; 2 Department of Psychology, Technical University of Dresden, Dresden, Germany; 3 Stadtjugendamt München (Child Protection Services Munich), Munich, Germany; 4 Amt für Jugend, Familie und Bildung Leipzig (Child Protection Services Leipzig), Leipzig, Germany; 5 Max Planck Institute of Psychiatry, 80804 Munich, Germany; 6 Munich Cluster for Systems Neurology (SyNergy), Munich, 81377 Germany; 7 University of Liverpool, Institute of Translational Medicine, Liverpool, L69 3BX UK

**Keywords:** Maltreatment, Developmental psychopathology, Internalizing disorders, Developmental pathways, Neuroendocrine functioning, HPA axis, Gene-environment interaction, Cognitive-emotional strategies

## Abstract

**Background:**

Effective interventions for maltreated children are impeded by gaps in our knowledge of the etiopathogenic mechanisms leading from maltreatment to mental disorders. Although some studies have already identified individual risk factors, there is a lack of large-scale multilevel research on how psychosocial, neurobiological, and genetic factors act in concert to modulate risk of internalizing psychopathology in childhood following maltreatment. To help close this gap, we aim to delineate gender-specific pathways from maltreatment to psychological disorder/resilience. To this end, we examine the interplay of specific maltreatment characteristics and psychological, endocrine, metabolomic, and (epi-)genomic stress response patterns as well as cognitive-emotional/social processes as determinants of developmental outcome. Specifically, we will explore endocrine, metabolomic, and epigenetic mechanisms leading from maltreatment to a higher risk of depression and anxiety disorders.

**Methods/design:**

Four large samples amounting to a total of N = 920 children aged 4–16 years will be assessed: Two cohorts with prior internalizing psychopathology and controls will be checked for maltreatment and two cohorts with substantiated maltreatment will be checked for internalizing (and externalizing) psychopathology. We will apply a multi-source (interview, questionnaires, official records), multi-informant strategy (parents, children, teachers) to assess maltreatment characteristics (e.g., subtypes, developmental timing, chronicity) and psychopathological symptoms, supplemented with multiple measurements of risk and protective factors and cutting-edge laboratory analyses of endocrine, steroid metabolomic and epigenetic factors. As previous assessments in the two largest samples are already available, longitudinal data will be generated within the three year study period.

**Discussion:**

Our results will lay the empirical foundation for (a) detection of early biopsychosocial markers, (b) development of screening measures, and (c) multisystem-oriented interventions in the wake of maltreatment.

## Background

Over 10 % of children in western societies experience maltreatment [1–8]. Of the many health risks exhibited by individuals with a history of childhood maltreatment [9–13], one of the most consistent is a marked rise in risk for emotional and internalizing symptoms and disorders (e.g. Posttraumatic Stress Disorder; PTSD; Major Depressive Disorder; MDD; Anxiety Disorders) [14–20]. Thus, Scott et al. [17] found that abuse or neglect reported to child protection services (CPS) amplifies risk 5 to 10-fold for PTSD, 2 to 2.5-fold for mood disorders, and 2.5 to 3-fold for anxiety disorders in adulthood. Nevertheless, maltreated children are a heterogeneous group and some develop alternate conditions, whereas others do not develop poor mental health at all (“multifinality”) [21]. Importantly, we know very little about such multifinality or its origins, especially in childhood and adolescence [16, 22]. The research project “Analyzing pathways from childhood maltreatment to internalizing symptoms and disorders in children and adolescents” (AMIS) seeks to shed light on the heterogeneity in response to trauma by assessing endocrine, metabolomic, and genomic stress response patterns to maltreatment alongside specific maltreatment characteristics, cognitive-emotional/social factors and psychopathological outcome in childhood.

### Theoretical background of our proposal

Aiming to illuminate multifinality following maltreatment, AMIS is rooted in theories on differential susceptibility and biological sensitivity to context [23], which claim that individuals differ systematically in their sensitivity to environmental inputs. Based on Bronfenbrenner’s [24] famous dictum that human development is a product of the exchanges between the individual and his/her environment, we assume that environmental influences (e.g., maltreatment) on developmental outcomes are modulated by genetic and neurobiological susceptibility to the environment [25], with gender potentially serving as a key moderator [26]. We seek to describe divergent developmental response profiles upon experiences of maltreatment by identifying different developmental pathways and outcomes. We firmly believe that a better understanding of multifinality soon after childhood maltreatment – which is the time when children often present to the Child Protective Services (CPS) – may benefit the development of effective individually tailored interventions.

We focus on the following putative key research areas (A. – E.) in AMIS to gain a more comprehensive understanding of developmental outcomes after maltreatment:

#### A. Pattern of childhood maltreatment

A well-documented source of heterogeneity in responses to maltreatment is the specific form and pattern of abuse and neglect a child experiences (e.g. subtype, severity, perpetrator, frequency, chronicity, and onset). For example, especially physical abuse which is chronic and neglect with early onset, predict internalizing and emotional symptoms in childhood and adolescence [16, 22, 27–30], giving rise to greater short- and long-term risk of internalizing problems [16, 28]. Moreover, two studies by Kaplow and colleagues [31, 32] document that substantiated maltreatment (neglect, sexual and physical abuse) occurring prior to age 5 predicts greater anxiety and depressive symptoms in adulthood than maltreatment at later stages. Thus, beyond the dichotomous question of whether maltreatment took place or not, it is crucial to determine precisely which subtype(s) occurred during which periods of life, who exposed the child to maltreatment and at what frequency. Importantly, exact chronicling of patterns of childhood adversity and maltreatment is a precondition for accurate estimation of the role of genetic, neurobiological and psychological factors in determining developmental outcome.

#### B. Genetic factors

Human behavior and molecular genetic data suggest that genetic makeup acts as a crucial determinant of developmental outcome. In keeping with estimates of the heritability of adult depression [33], twin-studies estimate that genetic factors explain up to 40-50 % of the risk for emotional disorders in childhood and adolescence [34–40]. Candidate gene studies have implicated several depression genes thought to be involved in central neurotransmission [41–45] (e.g., SLC6A4, TPH2, GRIK3, and P2RX7), neurotrophic factors [46, 47] (e.g., BDNF and DISC1) and stress response regulation [48–50] (e.g., ACE and NR3C1). However, with a few notable exceptions [51], the role of these genes have not been confirmed via replication or meta-analysis and in a recent mega-analysis no single-nucleotide polymorphism crossed the boundary for genome-wide significance [52].

Accordingly, scholars contend that while genetic factors are important, they do not operate in isolation, but rather determine resilience/vulnerability jointly with a number of other variables, including environmental characteristics [53]. A series of landmark studies of gene-environment interaction (GxE) helped to nurture a growing appreciation that specific genetic variants confer heightened susceptibility to psychopathology, but merely if individuals also experience environmental adversity [54–57]. Yet, despite these promising early results for single genotypes, many more genetic or environmental factors are likely involved in moderating short or long-term responses to stressful life events [57, 58], possibly accounting for failures to confirm these early findings in recent meta-analyses [59, 60].

These developments have prompted researchers to broaden their focus to include groups of genes known to be involved in the neurobiological stress response systems, such as the hypothalamic-pituitary-adrenocortical (HPA) axis. Thus, several labs have now observed GxE for variants of the CRHR1 gene, encoding the main receptor for the stimulant effects of the hypothalamic neuropeptide CRH on the endocrine stress response system [61–64]. Findings in adults also converge on FKBP5, which expresses an important modulator of the glucocorticoid receptor (NR3C1) involved in the regulatory feedback loop of the HPA axis. Specifically, a particular variant of this gene contributes to both responses to acute standardized stress [65] as well as development and relapse rate of internalizing disorders [66, 67]. Importantly, recent work from one of our labs indicates that a specific susceptibility genotype of FKBP5 confers elevated vulnerability to depression in young adults exposed to earlier critical life events while protective effects ensue for those with few or no such life events [68]. These data now clearly mandate research extending these patterns to childhood maltreatment and developmental samples.

#### C. Endocrine stress response

Over the past three decades evidence has accumulated that childhood maltreatment and adversity may exert marked and persistent influence on HPA axis activity [69–72]. These changes, in turn, constitute a risk factor for the development of internalizing and externalizing disorders in childhood and adulthood with gender-specific pathways [26, 71, 73–79]. Ample data from our labs and others implicate altered cortisol secretory activity as a correlate of childhood maltreatment, irrespective of whether cortisol levels were assessed during infancy [80], childhood [69, 81–83], adolescence [84] or adulthood [73, 85–88]. Interestingly, while aberrant cortisol secretory patterns have been repeatedly documented in maltreated subjects, some variability in the direction of reported findings has emerged [71, 89]. For example, whereas elevated cortisol concentrations were found in 24-h urinary as well as in morning salivary samples of maltreated children [69, 84], maltreated children also have also been found to exhibit attenuated morning salivary cortisol concentrations [83].

These inconsistencies may partly originate from lack of research integrating HPA axis findings with data on genotypes, epigenetic programming as well as psychosocial processes. In addition, variations in maltreatment experiences themselves may account for some of these inconsistencies. Research from our group [88], for example, shows that in a sample of 623 international adult adoptees, those with early experience of severe neglect exhibit lower morning cortisol levels (hypocortisolism) and a flatter cortisol decline across the day vs. non-abused adoptees. Conversely, neglected adoptees who also experienced moderate physical abuse displayed higher morning cortisol and a steeper diurnal decline. Other studies also report fluctuations in cortisol patterns as a function of maltreatment subtype [21].

Moreover, divergent, partly gender-specific, developmental pathways and mental disorders following trauma may give rise to differential cortisol patterns. For example, hypocortisolism and blunted cortisol responsivity typifies individuals with PTSD and/or exposed to traumatic life events [76, 90–93] (with some inconsistencies [71, 94, 95], possibly related to recency of trauma [96]), while a hyperactive HPA axis often accompanies adult depression [71, 97–99], a pattern considered especially characteristic of females [71]. Further findings in school-age children [69, 81, 82, 100] also suggest that mainly the combination of internalizing symptoms and maltreatment coincides with HPA axis dysregulation (e.g. flatter diurnal decline) while either one of these alone yields cortisol patterns comparable to controls.

As well, most stress response studies focus on glucocorticoids as effector hormones of the HPA axis. However, glucocorticoids are part of a balanced metabolic cascade, and homeostasis of the glucocorticoid metabolism is required for a fully functional stress hormone regulation [101, 102]. This calls for assessments at various tiers of the metabolic cascade to gain a fuller picture of the trauma-related anomalies in neuroendocrine functioning.

Finally, previous research has often set out to examine trait-like long-term patterns in cortisol secretion (i.e., modulation of allostatic set-points; [101]). However, obtaining valid estimates of long-term cortisol levels has been methodologically challenging using assessments in blood, saliva or urine which reflect short-term hormone levels and may suffer from measurement error and situational confounding [103–105].

Meeting the need for a measure of longer-term cortisol secretion, one of our labs has pioneered the measurement of cortisol in hair (see [106]). As cortisol is assumed to be incorporated into the hair shaft during hair growth, the examination of cortisol in a specific hair segment should provide a retrospective index of cumulative cortisol secretion over the time period during which the hair segment has grown [107, 108]. Recently, promising results link hair cortisol levels to differential levels of stress-exposure in adolescents [109] as well as internalizing disorders in adults [77, 93, 99]. However, little or no work to date extends these findings to younger children or well-characterized maltreated populations. More generally, work on HPA axis functioning lacks large-scale investigations of developmental samples, integrating neuroendocrine measures with genetic and psychological data.

#### D. Epigenetic programming

Animal studies and an increasing number of human findings now suggest that epigenetic, experience-dependent modifications of genes related to stress response regulation are involved in long-term imbalance of the HPA axis and may thereby sculpt developmental pathways. In their seminal work, Weaver and colleagues [110] demonstrated that maternal caregiving behavior in rodents (e.g., licking, grooming, arched-back nursing) affected hippocampal glucocorticoid receptor (GR) function via epigenetic programming including DNA methylation and histone modification. Unless treated pharmacologically, these effects persisted into adulthood with long-term impact on stress response and behavior. Likewise, differences in N3RC1 promoter methylation and corresponding changes in glucocorticoid receptor mRNA were found in post-mortem hippocampal sections of suicide victims, which varied as a function of experienced childhood abuse, thus suggesting a parallel effect of early parental care on epigenetic regulation of GR expression in humans [111]. Recent studies point to genotype-specific role of the DNA methylation in the FKBP5 gene with respect to infant neurobehavioral development [112] and risk for adult stress disorders [113]. As NR3C1 and FKBP5 are key modulators of the stress response and HPA axis regulation, epigenetic programming linked to early adversity may be of special importance for development of internalizing disorders, in turn, effecting changes in the HPA axis, among others [114–116]. Yet, little work in humans examines these epigenetic mechanisms as they take shape during ongoing development.

#### E. Psychological processing of maltreatment and social support

Psychological processing of early adversity and the individual social support available to the child, may also explain unique variance in the diverse pathways of risk [117], including the dysregulation of the HPA axis following deleterious rearing experiences [100]. For example, a study by one of our labs [118], supports the notion that it is the child’s *representation* of caregivers rather than caregiving environment per se that gives rise to elevated risk of behavioral symptoms in preschoolers. Indeed, recent neural data suggest that insecure attachment representations of caregivers also give rise to negative expectations during future interactions with unfamiliar peers, thus providing first empirical support to the neurocognitive mechanism driving the influence of representations across development [119, 120]. Further studies of ours [121–124] and other groups [125, 126] suggest that narrative techniques are appropriate from preschool age and aid in the detection and prognosis of gender-specific cognitive emotional styles that act as risk/protective factors for behavioral and internalizing symptoms. A number of groups [127–132] have identified ample cognitive emotional styles in maltreated children (e.g., dissociation, negative caregiver representations). In turn, these factors partly mediate effects of maltreatment on the level of social competence [133] and behavioral symptoms [134], underscoring their influence on developmental pathways.

#### F. Multiple perspectives on developmental outcome

Finally, when mapping pathways, it is crucial to assess mental health outcome in children in a highly accurate manner. To date, there has been a strong reliance in the maltreatment literature for young children on brief questionnaires, often completed by single external informants (e.g. parents). Data from one of our labs [135] and others [136, 137] demonstrate that is possible to maximize accuracy in outcome assessment by sampling multiple psychological perspectives on mental health problems, including those of children, parents, and teachers.

### Study aims

Owing to the aforementioned gaps in the evidence base we seek to:

1. describe and quantify different patterns/clusters of children with maltreatment experiences and/or psychopathological symptoms (e.g., internalizing, externalizing, healthy), drawing on a multi-method, multi-informant approach [136] to assess maltreatment and psychopathology via age-appropriate, reliable and valid interview, self-, parent- and teacher-reports;
2. identify factors that predict mental health problems, especially internalizing symptoms: duration, severity, quality, and time of maltreatment, family/social support, cognitive emotional styles, and genetic variants, especially in genes previously identified as potential susceptibility factors for internalizing disorders; farther, we aim to evaluate effects of dysregulation of the neurobiological stress system in terms of cortisol secretion;
3. evaluate the interaction of these factors, especially the mediating role of the neurobiological stress system and the roles of gender and genetic variants in stress-response related genes as moderators of associations between maltreatment experiences and internalizing symptoms;
4. explore possible etiopathological mechanisms in a subgroup of children related to altered steroid metabolism or epigenetic modification leading from adverse maltreating environment to emergence of psychopathological (especially internalizing) symptoms and disorders.

## Methods/design

### Samples

We will draw on two existing large-scale samples in Leipzig (Samples 1 and 2) and two additional samples (Samples 3 and 4), recruited expressly for AMIS from child protection services (CPS) Leipzig and Munich. Leipzig is a city in eastern Germany, with a population of 500,000, and above-average rates of families living in poverty. Munich is a metropolis (1.35 million inhabitants) in southern Germany with less poverty relative to Leipzig, but a high number of immigrant families.

Sample 1: For the current project, we aimed to remobilize ~ 90 % of a sample of 251 children originally oversampled for internalizing symptoms at preschool age [138]; first assessment funded by the German Research Foundation, KL 2315/1-1). Also, we sought to recruit an additional 75 children for a second wave of basic data-collection yielding a total subsample of N = 300. Children will be aged 4–8 years. Relevant variables/information were collected prior to AMIS (e.g. internalizing symptoms and disorder, saliva cortisol under stress conditions, genotyping).

Sample 2: Within AMIS we also aimed to reactivate roughly 60 % of a sample of 751 children, yielding a total subsample of N = ~450 children. This sample was originally comprised of 285 clinically referred children and 466 children from the general population, as part of the “B4a – LIFE Child Depression” cohort of the “Leipzig Research Center for Civilization Diseases, University of Leipzig” (LIFE, funded by the European Union, by the European Regional Development Fund/ERDF and the Free State of Saxony within the framework of the excellence initiative). Various data were collected at a first assessment (e.g., comprehensive diagnostic assessment, genotyping, see Tables 1 and 2) between 2011 and 2014. For AMIS children will be aged 9–16.

**Table 1:** Assessment of trauma/maltreatment, psychopathology, psychosocial factors, and physical development

Variables	Measures	Source	Informants	Age groups		Samples
**Child abuse and neglect**				4-8	9-14	
*Severity, subtypes, timing, frequency, perpetrator*	● MCS	Official documents	CPS file	x	x	3,4
	● CTS-PC	Interviews	Caregiver	x	x	1-4
	● MNBS	Questionnaires	Caregiver, Child		x	1-4
		Interviews (Picture-based)	Child	x		1-4
**Psychopathology**						
*General Symptoms*	● CBCL	Questionnaire	Caregiver	x	x	1-4
*(dimensional)*	● SDQ	Questionnaire	Caregiver, Child,	x	x	1-4
			Teacher	x	x	1-4
	● YSR	Questionnaire	Child		x	2-4
	● BPI	Puppet Interviews	Child	x		1, 3, 4
*Internalizing Symptoms*	● CES-DC	Questionnaire	Caregiver, Child	x	x	1-4
*(dimensional)*	● SCARED	Questionnaire	Caregiver, Child	x	x	1-4
	● CHILD-S	Questionnaire	Caregiver	x	x	2-4
	● ETI-CA	Questionnaire	Caregiver, Child	x	x	1-4
*Psychiatric disorders*	● K-SADS-PL	Interview	Caregiver	x	x	2^a^, 3, 4
*(categorical)*	● PAPA	Interview	Caregiver	x	x	1^a^,3
**Child environment**						
*Social support*	● ASSIS	Interview	Child, Parent	x	x	1-4
*Peer environment*	● PVS	Questionnaire	Child, Teacher	x	x	1-4
*Family environment*	● FES	Questionnaire	Caregiver	x	x	1-4
	● CTQ	Questionnaire	Caregiver	x	x	1-4
	● CIPA	Questionnaire	Caregiver	x	x	1-4
	● APQ	Questionnaire	Caregiver	x	x	1-4
*Caregiver factors*	● PHQ	Questionnaire	Caregiver	x	x	1-4
	● CTQ	Questionnaire	Caregiver	x	x	1-4
	● BSSS	Questionnaires	Caregiver	x	x	1-4
*Overall Adversity*	● ETI, ETI-KJ	Questionnaire, Interview	Child, Caregiver	x	x	1-4
	● FAI	Questionnaire, Interview	Child, Caregiver	x	x	1-4
**Psychological factors**						
*Emotion regulation, child representations of caregiver*	● NIT	Child narratives (Video Coding)	Child, parent	x	x	1-4
*Temperament*	● CBQ	Questionnaire	Caregiver	x	x	1-4
*Self efficacy*	● SPPC-D	Questionnaire	Child		x	1-4
*Ego-resiliency*	● ERS	Questionnaire	Caregiver, Teacher	x		1-4
*Psychological stress-response*	● TSST-C	Questionnaire			x	2,3
*Social Competences*	● SOCOMP	Questionnaire	Teacher	x	x	1-4
*Callous Unemotional traits*	● ICU	Questionnaire	Teacher	x	x	1-4
**Physical development**						
*Body-Mass-Index*	● Height	Measurement	-	x	x	1-4
	● Weight	(Measuring tape/scales)				
*Pubertal development*	● Tanner Scales	Questionnaire	Child		x	2-4

N.B. APQ = Alabama Parenting Questionnaire, ASSIS = Arizona Social Support Interview Schedule, BPI = Berkeley Puppet Interview, BSSS = Berlin Social Support Scales, CBCL = Child Behavior Checklist, CBQ = Children’s Behavior Questionnaire, CES-DC = Center for Epidemiological Studies Depression Scale for Children, CHILD-S = Children’s Depression Screener, CIPA = Coparenting Inventory for Parents and Adolescents, CTQ = Childhood Trauma Questionnaire, CTS-PC = Parent–child Conflict Tactics Scales, ERS = Ego resiliency Scales, ETI = Essener Trauma Inventar, ETI-CA = Essen Trauma-Inventory for Children and Adolescents, FAI = Family Adversity Index, FES = Family Environment Scales, ICU = Inventory of Callous-Unemotional Traits, K-SADS-PL = Schedule for affective disorders and schizophrenia for school-age children present and lifetime version, MCS = Maltreatment Classification System, MNBS = Multidimensional Neglectful Behavior Scale, NIT = Narrative Interaction Task, PAPA = Preschool Age Psychiatric Assessment, PHQ = Patient Health Questionnaire, PVS = Peer Victimization Scale, SCARED = Screen for Child Anxiety Related Disorders, SDQ = Strengths & Difficulties Questionnaire, SOCOMP = Self and Other oriented Social Competences, SPPC-D = Self-Perception Profile for Children, SSR-DE = Deutsche Version des Sleep Self Report, TSST-C = Trier Social Stress Test for Children, YSR = Youth Self-Report

^a^Diagnostic interviews in these samples were collected prior to AMIS

**Table 2:** Assessment of child neurobiological and genetic factors

Variables	Source	Measure	Assessment	Age groups		Samples
**Neurobiological factors**				4-8	9-14	
*Chronic stress exposure*	● Hair	● Cortisol	Normal appointment	x	x	1-4
*Genomic mechanisms*	● Plasma/Saliva	● Steroid metabolomics	Trier Social Stress Test for Children (TSST-C)		x	2^a^,3^a^
		● Methylation				
*Stress response regulation*	● Saliva	● Saliva cortisol	TSST-C		x	2^a^,3^a^
	● Plasma	● Blood cortisol				
		● α-Amylase				
**Genomic factors**						
*Susceptibility genes*	● Saliva/Plasma	● Focus on HPA-axis-related genes	Normal appointment	x	x	1-4

^a^TSST-Cs will only be conducted in a subsample of N = 80-100 children

Sample 3: We will recruit N = 150 maltreated children and their caregivers, receiving interventions from the Leipzig Child Protection Service (CPS) due to maltreatment, including those taken into custody in accordance with the law (§ 42 SGB VIII; Age range: 4–16 years).

Sample 4: 70 maltreated children, aged 9–14 years with severe maltreatment will be recruited using a comparable protocol as under 3) by the Munich CPS.

Prior research on maltreatment in these age-groups (<16 years) [1–4, 6–8, 22, 139–141] leads us to expect a balanced gender distribution in all samples, but higher incidences of (a.) sexual abuse in girls and (b.) overall maltreatment in our clinically referred sample.

#### Inclusion criteria for all samples

Participation is conditional upon the informed consent from legal guardians and assent of the child. To be eligible for the study, caregivers and children must speak German at a sufficient level and children’s cognitive development must be within the normal range (i.e., excluding severe mental impairment) as deficits in these areas may otherwise interfere with the comprehension and completion of measures.

#### Compensation for participation and sample maintenance

Caregivers will receive monetary compensation to take part in the study. Compensation will vary with the extent of time required for the investigations. Children will receive a small gift for their participation. Previous research experience shows that compensation is imperative for the establishment and maintenance of developmental samples. In order to maintain contact with families prepared to participate, measures for sample maintenance will be implemented (e.g., Birthday and Christmas greetings to the child, postcards to inform us of changes in address or contact details, etc.).

#### Recruitment procedure

Recruitment of most families in Samples 1 and 2 took place as part of previous research. Additional families in Sample 1 will be recruited through two channels: (1.) routine medical check-ups of the Health Department of Leipzig assessing school readiness and (2.) via ads and flyers in kindergartens. Caregivers will be informed about the study and invited to participate. Should families express interest to take part, they will receive written information about the project along with consent forms. An appointment for the investigation will be arranged via phone and confirmed a few days in advance.

Research fellows situated within the CPS will be in charge of recruitment of Samples 3 and 4. These fellows will select and contact participants in accordance with the inclusion criteria via CPS social workers. If necessary, joint home visits of the project associate and the CPS worker will take place in selected families to provide oral and written information concerning the research project. If caregivers and children agree to participate, written consent for participation and access to CPS records will be ascertained. Subsequently, appointments with caregivers and child will be arranged.

#### Data-collection

For Samples 1 and 2, comprehensive data from a first wave of data-collection will be available. Additional assessments will take place in these samples. In brief, the following types of data will be collected in the respective samples:

Sample 1: Caregivers and teachers will complete questionnaires while caregivers and children will be interviewed to assess maltreatment experiences, social support, emotion regulation (see Tables 1 and 2). Also, we will collect hair samples from each child (time expenditure: 2 h individual session with caregiver and child, in parallel).

Sample 2: Children, caregivers, and teachers will complete questionnaires and/or interviews to assess the same constructs as in Sample 1 (see Tables 1 and 2). Hair samples of each child will also be collected (time expenditure: 2 to 3 h individual session with caregiver and child, in parallel). A subsample of Sample 2 (n = 25, plus n = 50 controls) will take part in an in-depth assessment of stress-regulation (time expenditure: 2.5-3 h).

Samples 3 and 4: Data-collection is comprised of two parts for all families. First, assessment of CPS records conducted by CPS research fellows (time expenditure: 3.5 h per family). Second, two appointments will take place with caregiver and child present. Caregiver and child will be seen in parallel and administered all age-appropriate measures in Samples 1 and 2, as well as clinical interviews. The time expenditure will amount to approximately 2.5-3 h per appointment.

We will videotape all caregiver and child interviews for assessment purposes. Diagnostic interviews will follow a standardized protocol. Hair samples and genetic data of the child will not be collected in Sample 4 in compliance with local legislation in Munich. Caregivers will be asked to complete questionnaires at home between the two appointments and return them to the AMIS team. Teachers of the child will be asked to complete teacher-reports which are sent to them by mail. Third, a subsample of sample 3 (n = 25; age 9–14 years) will be invited to take part in an in-depth assessment of stress-regulation (time expenditure: 2.5-3 h).

### Ethics approval

The study has been approved by the respective ethics committees of the Universities of Leipzig and Munich, Germany (registration numbers for Leipzig Samples 1 and 2 178-12-21052012, Leipzig Sample 3, 098-12-05032012, and Munich Sample 4 098-12-05032012). Parents are informed orally and in written form about the contents and aims of the study and must give their written consent in order to enroll. All participation is voluntary and may be withdrawn by the family at any time without penalty or consequences of any kind. All procedures are in accordance with the Helsinki declaration. For more intensive neurobiological assessments (e.g., blood draws during stress protocol) numerous safety measures have been put in place (e.g., exclusion of children with acute and severe psychopathology; positive feedback to children at the end of the task; children are accompanied by a caregiver to and from the lab).

### Measures

Detailed descriptions measures will follow below, with an emphasis on key novel instruments (see Table 1). We only give cursory attention to widely used and well-known measures or assessments secondary to the main hypotheses. In the following sections, we refer to any responsible adult who has had close and regular contact to the child for a significant amount of time as “caregiver”. For data-acqusition in AMIS, this person acts as the primary external informant as many children will not be residing with their biological parents.

#### Patterns of childhood maltreatment and adversity

It is very challenging to operationalize maltreatment for several reasons: due to social stigma attached to it, because it usually occurs in private (i.e. without external observers), and as disclosure may lead to severe consequences [142]. Bearing these difficulties in mind, we will ascertain characteristics of maltreatment with a comprehensive, multi-source (interviews, self- and caregiver-reports), multi-informant (child, parent) strategy in all samples to minimize false-negative cases, in particular. For samples 3 and 4, we will also conduct an intensive standardized evaluation of CPS records, respectively, as is common practice in this field (3.5 h per record). In cases of new disclosures that require immediate action due to ongoing risk to the child, we have put measures in place (e.g., reporting cases to CPS or for cases known to CPS, intensifying interventions, if necessary).

Interviews with the caregiver and analyses of CPS records will be rated using the Maltreatment Classification System (MCS) [143] – a highly accurate, comprehensive, widely used and validated standardized system to evaluate maltreatment events reported in CPS records and caregiver interviews, based on the Maternal Maltreatment Classification Interview (MMCI) [144]. The MCS will serve as the basis for our assessment of case records (CPS) as well as our interviews to optimize comparability between our different samples and to ongoing international research. The MCS distinguishes between all major subtypes, i.e., neglect, including failure to provide and lack of supervision, sexual, physical, and emotional maltreatment (the latter includes witnessing parental violence), describes anchor examples, and formulates inclusion and exclusion criteria for each of these. Also, all relevant dimensions of maltreatment (subtype, severity, frequency/chronicity, developmental period, separations/ placements, perpetrator) are coded for each incident. One of the authors (Jody T. Manly, PhD) provided on-site training and helped to adapt the MCS to a German environment. Interviews with caregivers of affected children last approximately 45–60 min, while interviews with caregivers of unaffected children usually last between 10–30 min.

Children will additionally be requested to self-report on their child abuse and neglect experiences, using an adaptation of the Multidimensional Neglectful Behavior Scale (MNBS) and the Parent–child Conflict Tactics Scale (CTS-PC), with pictorial versions for children ≤8 years (15–20 min. each) [145, 146]. Further, we will administer the Essen Trauma-Inventory for Children and Adolesents (ETI-CA) [147, 148] and the Family and Life Events Sections of the Preschool Age Psychiatric Assessment (PAPA) [149, 150] to all caregivers and older children (≥9 years) to evaluate presence of critical life events.

#### Psychiatric symptoms and diagnoses

##### Psychiatric symptoms

We will quantify psychiatric symptoms using a multi-method, multi-informant approach, drawing on caregiver-, teacher- and child-ratings via reliable and valid questionnaires and interviews as well as age-appropriate puppet interviews [136]. To this end, caregivers and teachers will complete the Strengths and Difficulties Questionnaire [151], a 25-item screening measure, with good rates of compliance from parents and teachers due to its brevity. The 25 questions encompass four symptom scales (behavior problems, emotional problems, hyperactivity, and peer problems) and prosocial behavior. To assess total difficulties the scores of the four symptom scales are summed. Additionally, caregivers will complete the Child Behavior Checklist (CBCL) [152], a robust and broadly validated caregiver-report instrument. The questionnaire encompasses symptom scales which are grouped under internalizing syndrome (Withdrawn, Somatic Complaints, Anxious Depressed) and externalizing syndrome (Rule-breaking Behavior, Aggressive Behavior). By using the existing German norms for these measures e.g., [153, 154], the scores can be categorized into three groups: normal, borderline, or abnormal.

As the focus lies on internalizing symptoms, all caregivers will additionally complete the 20-item Center for Epidemiological Studies Depression Scale for Children (CES-DC) [155] and the 41-item Screen for Child Anxiety Related Emotional Disorders (SCARED) [156]. Further, we will administer the Essen Trauma-Inventory for Children and Adolesents (ETI-CA) [147, 148] to all caregivers and older children (≥9 years) to gauge PTSD symptomatology.

Children aged nine and above will complete a number of self-reports. First, we will collect the child-adapted version of the SDQ and the Youth Self Report (YSR) which assesses the same dimensions as the CBCL [157, 158]. Second, children in this age band will also complete the child versions of the CES-DC, SCARED and ETI-CA as well as the Children’s Depression Screener (ChilD-S) [159].

Children aged 8 and younger will be requested to complete the Berkeley Puppet Interview (BPI) [137, 160] to assess children’s self-perceptions of symptoms. The BPI blends structured and clinical interviewing techniques to elicit the child’s own perspective of their strengths and difficulties. Experimenters conduct interviews with the help of two identical hand puppets that make two opposing statements on a topic before asking children to describe themselves. Independent raters will score interviews from videos. The BPI has undergone extensive testing for reliability and validity with preschoolers e.g., [137, 161]. Each interview lasts 30 min. Interviewers and coders receive intensive on-site training.

##### Psychiatric disorders

Internationally well-established structured diagnostic interviews will be employed to derive concurrent categorical diagnostic classifications in Samples 3 and 4. They will parallel those already collected in Samples 1 and 2: the Preschool Age Psychiatric Assessment (PAPA) [149, 150], for younger children in Sample 3 and the Schedule for Affective Disorders and Schizophrenia for school-age children, Kiddie-SADS-Present and Lifetime Version (K-SADS-PL) [162] in Samples 3 and 4. The PAPA is a comprehensive, structured, glossary-based psychiatric interview for assessing psychiatric symptoms, symptom scale scores, and diagnoses, as well as life events, family structure and functioning, and impairment in children aged 2 to 8. The PAPA provides a standardized measure of DSM-IV psychiatric symptoms and disorders in preschoolers. An electronic version of the PAPA, the ePAPA, enables administration by touchscreen-based laptops. All relevant data are recorded directly in the ePAPA interface, allowing for fast, automated assessment. Research assistants will conduct interviews with caregivers, lasting approximately 2.5 h, but may take longer in cases with high symptom-load.

The K-SADS-PL is a semi-structured interview based on DSM-IV and assesses current and lifetime history of psychiatric disorders of children. The K-SADS-PL consists of a screening interview and six diagnostic supplements. All participants are initially screened, followed by supplemental questions when screened positive. The interview will be conducted with caregivers, lasting approximately 2 h. Trained members of one of our labs have extensive experience using and coding the PAPA and K-SADS-PL. They will provide training to all AMIS staff using these interviews in Samples 3 and 4.

#### Psychological measures

We will assess cognitive-emotional styles of children with self-reports and narrative techniques (often used to study psychological processing of trauma) [30, 117, 127, 130, 131]. Teachers and parents will complete the Ego Resiliency Scales [163]. Also, parents will be enquired about their child’s temperament using the Children’s Behavior Questionnaire [164], and all children will be asked to complete Harter’s Self-Perception Profile for Children (SPPC) [165] to assess their self concept. Teachers will also complete the Inventory of Callous Unemotional Traits [166, 167].

Narrative techniques, in particular, have aided in the identification of gender-specific patterns and risk/protective factors for behavioral symptoms in children [118, 121–123, 125–127, 130, 131] as well as abnormal cognitive-emotional styles in maltreated samples, e.g.[127–133]. We will use a narrative interaction task informed by the MacArthur Story Stem Battery (MSSB) [168] and narrative-co-construction techniques. Children’s narrative responses following six story stems will be videotaped and rated with codes based on the MacArthur Narrative Coding Manual (MNCM) [169] and the Process Scales [170]. The narratives will be coded for content (e.g. negative representation of mother) and structure (e.g. coherence, denial, intentionality). The procedure is suitable for children age four and above. The procedure takes about 20 min with an additional 30 min required for coding.

#### Measures of social relationships

##### Social support

We will administer the Berlin Social Support Scales (BSSS) [171] to parents as well as Barerra’s [172, 173] Arizona Social Support Interview Schedule (ASSIS) to parents and children – a relatively brief interview (20–30 min.) previously used in children as young as 5 years [55, 174]. It operationalizes a child’s social network by requesting, for example, whom he/she can confide in when he/she wants to talk about personal issues or count on to purchase needed materials/objects. Next, the child rates the extent of contact he/she has to each social support and how much this person is perceived as caring. The child version of the ASSIS has proven highly successful in identifying the protective effects of social support: (a.) for childhood depression [173], (b.) for the combined negative impact of child depression and maltreatment on HPA axis dysregulation [100] and (c.) for children who carry risk genotypes for child depression following maltreatment [55, 174].

##### Socioeconomic burden

Socioeconomic status will be indexed by the Winkler Index [175], which takes into account educational as well as occupational status and income of caregivers. We will also use the Family Adversity Index (FAI) [176], which assesses important risk factors, such as low parental education, overcrowding in the family, persisting parental discord or one-parent family situation, maternal psychopathology, delinquency of the father, institutional care of the child exceeding one week in duration. The presence of each item is scored as 1 point and the total number is summed. The FAI has been of considerable importance in showing the link between family stressors and disorder in the child.

##### Family life

The Alabama Parenting Questionnaire (APQ) [177, 178] will be adminstered to assess six parenting constructs: (1) Parent Involvement, (2) Positive Parenting, (3) Poor Monitoring/Supervision, (4) Inconsistent Discipline, (5) Corporal Punishment, and (6) Other Discipline Practices. Parents and older children will serve as informants.

The quality of family relationships will be assessed via parental ratings using the German version of the Family Environment Scale (FES) [179, 180]. We will use the average score of the subscales cohesion, expressiveness and conflict to establish the quality of family relationships.

#### Neuroendocrine biomarkers of stress and stress response regulation

We have chosen two strategies to assess HPA axis functioning. These include (i) the innovative approach of measuring cortisol concentration in hair as a biomarker of chronic stress exposure and (ii) a well- established protocol to assess acute cortisol stress reactivity under standardized conditions (see Table 2).

##### Hair cortisol concentration (HCC)

An important aspect of the current proposal is the assessment of stable changes in cortisol secretion which are influenced by maltreatment in early childhood and persist throughout adolescence and adulthood. The assessment of cortisol in hair constitutes an innovative method which is assumed to provide an index of stable, long-term cortisol secretion. As cortisol is assumed to be incorporated into the hair shaft during hair growth, the concentration of cortisol in a specific hair segment should provide retrospective index of cumulative cortisol secretion over the specific time period of hair growth [107, 108]. Hair strands of a diameter of approximately 3 mm will be taken scalp-near from a posterior vertex position using fine scissors. Steroid concentrations in the two 3 cm hair segments most proximal to the scalp will be determined. Based on a hair growth rate of ~1 cm/month [181], these hair segments are assumed to represent cumulative steroids secreted over the 6-months period prior to hair sampling.

Over the past decade, the use of hair cortisol as an index of long-term cortisol secretion has been supported by considerable evidence, indicating both high test-retest reliability [182] and general validity of the method [183–188]. In addition, evidence has confirmed marker qualities of hair cortisol levels with regard to chronic psychosocial stress [189–192] as well as in the context of stress-related diseases [193–195]. Furthermore, hair cortisol has proven relatively robust to a range of potential hair-related confounding influences including dyeing, bleaching, straightening or permanent waves; [185, 196–198], though hair washing frequency and chemical hair treatments should be assessed and controlled for [199]. Besides hair-related parameters, it has been shown that age [200] and body fat-related measures [106, 201] may be associated with hair cortisol levels and should be considered as potential covariates.

##### Stress response regulation

To assess Salivary and plasma cortisol levels in response to a standardized stressor, we will administer the Trier Social Stress Test (TSST) [202–204] in its child-adapted form for Children (TSST-C) [204] to a subset of Samples 2 and 3 (n = 80-100; 9–14 years). The sample will comprise children with internalizing symptoms or disorders and a history of maltreatment as well as non-maltreated controls.

The TSST is a standardized protocol that has been found to produce the most significant and reliable HPA axis activation under laboratory conditions in a meta-analysis [205]. It is a resource-intensive procedure with a duration of about three hours, requiring participation of up to four staff members. Besides measures of the HPA axis, we will analyze salivary amylase activity as a proxy of sympathetic activation [206] as well as steroid metabolomic factors and gene methylation (see below).

##### Steroid metabolomics

Change in cortisol levels is the most accessible read-out of the human stress response. However, cortisol is embedded in complex metabolomic processes limiting the information that can be provided by a single element of the steroid pathway [207]. In this study we are planning to measure additional steroids to obtain a complete picture of the corticosteroid pathway. It can be assumed that a disturbed balance of the steroid pathway is a major contributing factor for the medium and long-term effects of stress including the risk of developing stress-related mental disorders. Therefore, we will investigate the effects of a disturbed steroid metabolism on risk of psychopathology by evaluating corticosteroid and neurosteroid metabolites from plasma samples obtained in response to the TSST. These analyses will include the assessment of the precursors and metabolites of cortisol including 11-deoxycortisol and cortisone, the mineralcorticoid aldosterone and its precursor steroids 11-deoxycorticosterone and corticosterone, as well as the neuroactive steroid hormones dehydroepiandrosterone (DHEA) and dehydroepiandrosterone-sulfate (DHEA-S) as well as its metabolite androsterone. In addition, we will evaluate the androgen steroids androstenedione, testosterone and dihydrotestosterone or the progestogens progesterone and 17a-hydroxyprogesterone as well as estradiol to obtain a complete picture of gender-dependent stress effects on the steroid metabolism. Steroid metabolomic analyses will be performed using liquid chromatography in combination with mass spectrometry (LC-MS/MS).

#### Genetic assessments

##### Genetic moderators

Genetic variations are further potential moderators for stress and disease risk. The analyses focus on HPA axis-related genes and will be conducted using the following procedures:

- Selection of candidate genes identified from previous genetic case/control studies of internalizing disorders (e.g., SLC6A4, TPH2, GRIK3, P2RX7, BDNF, DISK1, ACE, NR3C1/GR, SLC6A15), from genetic stress response and gene-environment studies (e.g., NR3C1/GR, NR3C2/MR, FKBP5, FKBP4, STAT5B, CRHR1), and from the results of a Gene Ontology analysis focusing on the GO term “regulation of response to stress” (GO 0080134).
- DNA extraction from salivary (Oragene sampling devices) or blood samples (EDTA whole blood) depending on the sample and age of children; DNA extraction will be performed using respective Puregene extraction kits (Gentra).
- high-throughput DNA genotyping (all samples, N = 850) using Illumina HumanOmniExpress BeadChip providing a SNP coverage with an average distance of 2.1 kb for 200 candidate genes selected from a) SNPs tagging regions with copy number variations (CNV) will also be included. Genotyping will be performed with an Illumina iScan System.

We expect 95 % of participants with central European ethnic background. Nevertheless we will perform analyses with and without ethnic minority populations.

##### DNA methylation

Given the prominent role of NR3C1 (GR) and FKBP5 for the stress response regulation, epigenetic assessment will be specifically focused on these two genes, but genes showing maltreatment interactions with respect to disease risk (Aim 3) will also be included. Epigenetic analyses will be conducted with genomic DNA collected from the participants of the TSST study. DNA will be isolated from salivary samples and from blood cells using Puregene (Gentra) extraction kits. Methylation patterns within the promoter regions of the selected genes will be quantified using bisulfite conversion with a Quiagen DNA methylation kit. Methylation-specific primers will be designed using the Methyl Primer Express Software (Applied Biosystems), and sequencing will be conducted using an ABI Prism 3700 capillary sequencer.

#### Study eligibility

To ensure sufficient comprehension of child assessments, a number of validated measures of IQ will be administered to children at their first assessment (i.e., Samples 1 and 2 already received their first assessment prior to AMIS). These include Raven’s Coloured or Standard Progressive Matrices (CPM; SPM) [208, 209] for younger (<8 years) and older children (≥8 years), respectively, as well as the Culture Fair Test 20-Revised (CFT 20-R) [210]. Likewise, linguistic competence will also be assessed at children’s first assessment additionally using either the Marburger Sprachverständnistest für Kinder (MSVK) [211] in Sample 1 or the Peabody Picture Vocabulary Test-Revised (PPVT-R) [212, 213] in all other samples.

### Data-analytic procedures

Data analyses will be based on models of developmental psychopathology [214–217] outlining direct and indirect effects of risk and protective factors on outcomes and how these factors are inter-related. We will test cumulative, mediating and moderating models.

Cumulative models imply, for example, that maltreatment and HPA axis dysregulation are additive risk factors in their effect on internalizing symptoms. Conversely, mediation implies, that, for instance, one risk factor (e.g., abuse) leads to another (e.g., altered gene expression) which, in turn, precipitates internalizing symptoms. Moderation indicates, for example, that a risk factor (e.g., genotype) exerts a weaker effect in the presence of another factor (e.g., social support) than in its absence.

With regard to the analytical framework for genetic analyses, we will mainly apply methods originating from linear models, (e.g., generalized linear models, generalized linear mixed models) where appropriate. Analyses will take a bi- and multivariate form, with testing based on likelihood-ratio and quasi-likelihood methods, depending on the specific structure of the data and verified by permutation and other resampling methods. These methods will allow appropriate control of type I error, also in the context of correlated outcomes and predictors. We will also correct for multiple testing via resampling. To this end, we aim to control for the family-wise type I error rate. The expected number N of permutations is a function of the asymptotic type I error α, for a given value of α, N is set to 100/α, i.e. for an α-value of α = 0.001 we will expectedly perform 100/[0.001] = 100,000 permutations. All these analyses will be conducted using R.

Beyond this, and in a more exploratory fashion, we will also perform mediation analyses, structural equation models (using Mplus and R), path analyses and methods from machine learning (e.g., support vector machines, again using implementations in R and MATLAB). The latter methods offer the most efficient solution for complex analyses, especially if types of data samples vary – as is the case in AMIS.

In a third analytic step, we also plan to use methods to delineate pathways leading from genome and trauma to emergence of psychopathology. Here, we will use both aforementioned approaches and methods aiming to infer causality from observational data, preferentially building on Janzing and Schölkopf’s [218] framework. In addition, we will search for gender differences in all our analyses as a possible moderator. Due to the partial heterogeneity in our samples, we will insert sample membership in all analyses as a control variable, to assess whether results are homogenous in all four samples.

#### Power calculation

To determine the required total sample size, we focused on Aim 3 addressing the moderating effects of genotype and gender on the association between maltreatment and disease risk. The core analysis for this aim is a gene-environment interaction analysis between the genetic variants and maltreatment with respect to the development of internalizing symptoms and disorders. We are intending to select 200 candidate genes, for which we assume an average number of five independent LD blocks within the genes, resulting in 1,000 independent tests. Further, we will perform separate analyses for those interactions with a significant gender effect. We assume that this will increase the number of independent analyses to 1.200. From this number we calculate a Bonferroni-corrected alpha error of .05/1.200 = 4.17e-5. Based on a total sample size of 850 individuals, we achieve a power estimate of larger than 0.90 for the gene-environment interaction term in case of an effect size of at least 3 % explained variance, even for genotypes with a minor allele frequency of 1 %, suggesting sufficient power to detect also small gene-environment interaction effects. Power calculation was conducted with Quanto Version 1.2.3. An additive mode of inheritance was applied, and quantitative variables were assumed for the environmental factor (maltreatment score) and the disease trait (internalizing symptoms).

## Discussion

Our protocol addresses several important areas pertinent to broadening our grasp of different pathways of mental development in the aftermath of maltreatment, including neurobiological, psychological, social, and genomic processes. Though some of these areas have already been studied individually in some detail, in most cases work is currently limited to adults (e.g., hair cortisol) or certain subsets of maltreated populations (e.g., few studies comparing HPA axis functioning in children with emotional abuse or neglect to controls), and small samples of maltreated children (e.g., cognitive-emotional styles). Most importantly, the current collaboration of experts within each of these fields offers the opportunity to move beyond studying these processes in isolation by evaluating how these processes interact with one another to potentiate or reduce risk of internalizing symptoms and disorders following childhood maltreatment.

The greatest challenge for longitudinal studies on consequences of maltreatment lies in the difficulty of recruiting a sufficiently large sample of affected children and families. The majority of affected families suffers poverty and is non-compliant and/or lacks motivation to participate in research. Notwithstanding, we intend to undertake a concerted effort to implement our research strategies by joining forces with the local youth welfare offices/governmental child protection services at the forefront of working with such families. It is therefore of vast strategic importance that we have enlisted the heads of CPS Leipzig and Munich with their practical and research background as members of our research group. This alliance will enable us to carry out our scientific evaluations in samples with a large variance of maltreatment experiences, ranging from relatively transient and mild to chronic and severe.

Although the collaboration with the directors and the staffs of the CPS ensures the feasibility of the study in these populations, we also clearly acknowledge that these assessments will require great efforts: motivating the participating families, many short-notice cancellations of appointments, re-invitations, sometimes extended or repeated visits.

### Limitations

First, in Samples 1 and 2, we will run a second wave of data collection. However, in Samples 3 and 4 only one cross sectional assessment will be possible during the three year funding period. Therefore we will mainly be able to identify risk patterns rather than longitudinal causal chains. For example, data-analysis will not enable us to differentiate whether certain forms of HPA axis dysregulation are caused by maltreatment experiences or by psychopathology or whether they are consequences of reciprocal transactional processes influenced by both environmental and individual aspects of development. In the same vein, our study will mainly inform us about the risk/protective effects of these factors on concurrent mental health, but these may differ in important ways from long-term risk/protective effects [219]. Nevertheless, our careful cross-sectional assessments will serve as a starting point for designing long-term research strategies within a prospective design. Second, samples will be heterogeneous with respect to age and problem levels, which may limit comparability, but will improve generalizability and sensitivity to detect differences and associations. Finally, as is common practice in the field, we focus on post-hoc assessments of maltreatment, i.e. after it has taken place. Hence, we will not be in an optimal position to analyze causes for maltreating behavior. Yet, our design takes us much closer in time to the phenomena in question vis-à-vis adult studies that usually rely on distal retrospective reports.

#### Implications

Many local and national projects strive to prevent and treat individual and social problems engendered by childhood maltreatment. A common limiting factor is that these efforts suffer from a lack of empirical support and coordination. The proposed project will lead to direct benefits in the field of personalized prevention. The results will extend the evidence base for practice guidelines applicable to all fields of child protection. They will help consolidate a catalogue of risk indicators (biological and psychosocial) that may be ascertained in standardized ways in cases of child maltreatment.

Moreover, there are great efforts to improve interdisciplinary collaboration between the CPS and medical institutions (e.g., paediatric hospitals, child psychiatric departments) to establish common standards in dealing with maltreated children and their families. By means of direct cooperation with the CPS, this protocol extends this collaboration to the field of scientific research in this country, raising the collaborative standards to new and higher levels.

Indeed, many countries have seen various attempts to establish standard procedures in the face of child maltreatment, but these efforts were often not optimally coordinated across different disciplines and often lack empirical basis. Improved knowledge on patterns of psychopathology in the aftermath of maltreatment, risk factors as well as protective factors, and how these factors interact will lay the basis for improved diagnostic standards which are applicable to the fields of social work/child protection as well as clinical medicine.

In sum, AMIS will contribute substantially to a growing cross-national research effort towards broadening our grasp of differential response profiles of children with a history of maltreatment. In turn, these data will eventually pave the way for more judicious clinical decision-making and elaborating interventions in the wake of maltreatment tailored to the specific needs of the individual child.
